# Beyond Drugs and Surgery: Superselective Adrenal Artery Embolization Redefines Primary Aldosteronism Management—A Systematic Review and Meta‐Analysis

**DOI:** 10.1155/ije/7534774

**Published:** 2026-07-22

**Authors:** Kimia Darmiani, Mehrad Zare, Alisa Mohebbi, Mohammad Mirza-Aghazadeh-Attari, Mohammad Ghasemi Rad, Afshin Mohammadi

**Affiliations:** ^1^ School of Medicine, Tehran University of Medical Sciences, Enghelab Square, Tehran, Iran, tums.ac.ir; ^2^ Universal Scientific Education and Research Network (USERN), Keshavarz Boulevard, Tehran, Iran, usern.tums.ac.ir; ^3^ Department of Radiology and Radiological Science, Johns Hopkins University School of Medicine, Baltimore, Maryland, USA, jhu.edu; ^4^ Department of Radiology, Division of Vascular and Interventional Radiology, Baylor College of Medicine, Houston, Texas, USA, bcm.edu; ^5^ Radiology Department, Faculty of Medicine, Urmia University of Medical Science, Urmia, Iran, umsu.ac.ir

**Keywords:** primary aldosteronism (PA), safety and efficacy, superselective adrenal artery embolization (SAAE)

## Abstract

**Background:**

Primary aldosteronism (PA) is a leading cause of secondary hypertension associated with increased cardiovascular and cerebrovascular risk. Superselective adrenal artery embolization (SAAE) has emerged as a minimally invasive alternative to adrenalectomy and mineralocorticoid receptor antagonists (MRAs). This systematic review and meta‐analysis evaluates the efficacy and safety of SAAE in PA management.

**Methods:**

We searched PubMed, Web of Science, Embase, and Cochrane Library through June 2026 for studies enrolling patients with PA treated by SAAE. Outcomes of interest were changes in blood pressure (BP), biochemical markers (serum potassium, aldosterone, renin activity, and aldosterone‐to‐renin ratio), clinical and biochemical response rates, daily defined dose change, and adverse events. Data were pooled using a random‐effects model.

**Results:**

Nineteen studies comprising 1242 patients undergoing SAAE met inclusion criteria. SAAE achieved substantial BP reductions: office systolic BP −19.71 mmHg (*p* < 0.001), office diastolic BP −10.91 mmHg (*p* < 0.001), home systolic BP −18.58 mmHg (*p* < 0.001), and 24‐h ambulatory systolic BP −14.57 mmHg (*p* < 0.001). Serum potassium increased +0.34 mmol/L (*p* < 0.001), aldosterone declined −46.72 ng/dL (*p* < 0.001), and ARR decreased −32.64 (*p* < 0.001). Complete clinical response occurred in 27% and partial response in 43%; complete biochemical success was achieved in 48% with partial success in 16%. Antihypertensive burden decreased (−0.52 daily defined dose; *p* < 0.001). Adverse events were predominantly minor (postprocedural pain 37.17%, low‐grade fever 8.55%); serious complications were uncommon (< 1%).

**Conclusions:**

SAAE is an effective and somewhat safe treatment for PA that achieves meaningful BP control, biochemical normalization, and reduced medication burden. It represents a valuable option for patients ineligible for or intolerant of surgery or MRAs.

## 1. Introduction

Primary aldosteronism (PA) represents one of the most clinically consequential forms of secondary hypertension, distinguished by the autonomous overproduction of aldosterone from one or both adrenal glands [1]. The clinical significance of PA extends far beyond its contribution to hypertension prevalence, as mounting evidence demonstrates that patients with this condition face a markedly elevated risk of cardiovascular and cerebrovascular morbidities that substantially exceed those observed in essential hypertension [2–4]. The therapeutic landscape for PA has been characterized by a binary approach that reflects the underlying pathophysiology and anatomical distribution of aldosterone excess. Unilateral adrenalectomy remains the definitive treatment for patients with unilateral pathologies, offering the potential for complete biochemical and clinical cure. However, this surgical approach is contraindicated or declined by a substantial proportion of patients due to surgical risk factors, patient preference, or the presence of bilateral adrenal disease. For these individuals, mineralocorticoid receptor antagonists (MRAs) have historically served as the cornerstone of medical management, providing pharmacological blockade of aldosterone’s deleterious effects at the tissue level [1].

Despite their established efficacy in reducing cardiovascular risk and controlling hypertension, MRAs are associated with significant limitations that compromise their real‐world effectiveness and patient acceptance [5]. The side effect profile of these agents includes dose‐dependent antiandrogenic and progestogenic effects such as gynecomastia, breast tenderness, menstrual irregularities, and decreased libido [6]. Besides lifelong usage indication, they carry risks of hyperkalemia and acute kidney injury, particularly in patients with underlying chronic kidney disease or those receiving concomitant renin–angiotensin system inhibitors [7, 8]. The cumulative impact of these adverse effects results in high discontinuation rates and suboptimal long‐term adherence, potentially leaving patients vulnerable to the continued cardiovascular consequences of untreated aldosterone excess.

The inherent limitations of current therapeutic options have catalyzed interest in alternative treatment modalities that bridge the gap between surgical and medical management. Adrenal artery embolization (AAE) has emerged as a promising noninvasive intervention that addresses the fundamental pathophysiology of PA by directly targeting the source of excess aldosterone production. This percutaneous procedure involves the selective occlusion of adrenal arteries using embolic agents such as anhydrous ethanol, resulting in controlled necrosis of hypersecreting adrenal tissue and subsequent normalization of the aldosterone‐renin axis [9, 10]. The technological evolution of interventional radiology techniques has enabled the development of superselective adrenal artery embolization (SAAE), which allows for precise targeting of dysfunctional adrenal tissue while preserving normal adrenal parenchyma [11, 12].

Despite these promising theoretical and preliminary clinical advantages, the evidence base SAAE remains fragmented and heterogeneous, limiting its integration into established treatment algorithms. High‐quality systematic evaluation of existing evidence is therefore essential to determine the true clinical utility of SAAE and its appropriate positioning within the therapeutic armamentarium for PA. This systematic review and meta‐analysis seeks to address these critical knowledge gaps by providing a comprehensive synthesis of available evidence regarding the clinical utility of SAAE in PA management. Through systematic evaluation of blood pressure (BP) reduction, biochemical normalization, and clinical response rates, this analysis aims to establish the therapeutic efficacy of SAAE across diverse patient populations. Additionally, comprehensive assessment of reported adverse events will provide crucial safety data to inform clinical decision‐making and patient counseling. By incorporating comparisons with MRAs, this meta‐analysis will position SAAE within the existing therapeutic landscape and provide evidence‐based guidance for its clinical application.

## 2. Materials and Methods

This systematic review and meta‐analysis was registered in advance on the Open Science Framework (OSF) at https://osf.io/bngaz/ (see Supporting Information 1). To perform this study, we adhered to the comprehensive guidelines outlined in the Preferred Reporting Items for Systematic Reviews and Meta‐analyses (PRISMA), and Search (PRISMA‐S) (Supporting Information 2).

### 2.1. Search Strategy

The literature search was conducted on June 2, 2026, targeting four databases: PubMed, Web of Science, Embase, and Cochrane Library (the search strategy is provided in Supporting Information 3). Two experienced reviewers independently formulated the search method. The methodologies were integrated, with a third reviewer intervening in the case of disagreement. The search technique utilized terms for PA and SAAE, along with relevant EMTREE and MeSH terms. No language restrictions were applied. Additionally, the reference lists of pertinent papers were reviewed to uncover any overlooked publications.

### 2.2. Eligibility Criteria and Study Selection

Both observational study designs (including cross‐sectional, case–control, and cohort studies) and trial studies (encompassing both randomized and nonrandomized controlled trials) were considered eligible for inclusion in this systematic review. Conversely, we excluded case reports and case series, editorial articles, commentaries, correspondence pieces, clinical guidelines, existing meta‐analyses, systematic reviews, narrative reviews, and unpublished gray literature not appearing in peer‐reviewed academic journals.

Studies were eligible if they enrolled at least five patients with a confirmed diagnosis of PA and used SAAE as the intervention for PA management. Additionally, studies were required to confirm PA status within the study and to report clinical outcomes used in this study (clinical or biochemical treatment response, BP assessment, and serum electrolyte and metabolite levels, etc.). Studies were excluded according to several criteria; investigations employing SAAE techniques that involved nonselective obstruction of multiple adrenal artery branches were omitted, as this represents a fundamentally different procedure that affects substantially more adrenal tissue branches containing nonpathological tissue compared to SAAE; publications released before 2010 were excluded owing to technique evolvement over the time.

Two experienced reviewers separately screened the search results to exclude nonrelevant studies. The reviewers’ disagreements were settled through dialog. A third reviewer also took part in the case of disagreement. To prevent including duplicate reports, the reviewers double‐checked the author names and nationalities of the included studies.

### 2.3. Risk of Bias Assessment

The methodological quality and risk of bias assessment for each included study were systematically evaluated using the Risk Of Bias In Non‐randomized Studies of Interventions (ROBINS‐I) tool, a comprehensive framework specifically designed for assessing bias in nonrandomized intervention studies. Two independent reviewers conducted separate evaluations to ensure objectivity and minimize assessment bias, with disagreements resolved through structured discussion and consultation with a third reviewer when necessary. The reviewers separately evaluated the possibility of bias in seven areas of confounding factors, patient selection, interventions classification, deviation from intended interventions, outcome measurement, and reported results selection.

### 2.4. Data Extraction

Two reviewers conducted data extraction independently. The data were cross‐checked for consistency, and a third reviewer was consulted in the case of disagreement. Detailed study characteristics, patient demographics, and intervention type (SAAE or MRAs) were extracted. To evaluate the BP changes more precisely, we utilized several measurements, including home, office, and ambulatory monitoring. Serum potassium, plasma aldosterone concentration, plasma renin activity, and aldosterone‐to‐renin ratio (ARR) were recorded as the biochemical indicators. To measure treatment burden, antihypertensive drug consumption was standardized to defined daily doses (DDDs). All clinical and biochemical outcomes were assessed based on the primary aldosteronism surgical outcome (PASO) criteria, an internationally validated consensus framework for standardizing treatment response evaluation. Clinical outcomes were categorized into three tiers (complete, partial, and absent success) based on systolic and diastolic BP readings, antihypertensive medication requirements, and achievement of normotension. Complete clinical success was defined as the achievement of normal BP off all antihypertensive medications, while partial clinical success included patients with substantial BP reduction on fewer medications or residual hypertension despite treatment. Biochemical outcomes were similarly stratified into complete, partial, and absent success categories based on normalization of serum potassium levels, plasma aldosterone concentration, plasma renin activity, and ARR. Complete biochemical success denoted normalization of all biochemical parameters without evidence of persistent or recurrent mineralocorticoid excess, whereas partial biochemical success indicated improvement in some but not all biochemical markers [13]. Finally, adverse events were extracted thoroughly and classified using the definitions given in each research by severity (major vs. minor). Utilizing double‐arm studies, SAAE and MRAs, data were extracted to reach a robust consensus regarding SAAE’s place alongside traditional management.

### 2.5. Data Synthesis

All statistical analyses were conducted using MedCalc Version 23.2 (MedCalc Software, Ostend, Belgium) and Stata Version 17.0 (StataCorp, College Station, TX, USA). Pooled mean differences with 95% confidence intervals (CIs) were computed for continuous variables, including changes in systolic and diastolic BP throughout office, home, and 24‐h ambulatory monitoring, as well as biochemical indicators (serum potassium, aldosterone, renin activity, and ARR). Prior to pooling, results were translated to a common metric in cases where studies reported outcomes in various units. Pooled proportions with 95% CIs were computed for categorical outcomes, such as complete and partial clinical or biochemical response rates and adverse event frequencies. Risk differences or mean differences were computed when direct comparisons between SAAE and MRAs were available. For results that could not be meta‐analyzed because of inconsistent reporting, narrative synthesis was used. A high statistical heterogeneity was defined as the *I* ^2^ value of > 50%. A *p* value of 0.05 was used as the threshold to determine the statistical significance.

## 3. Results

### 3.1. Study Selection and Characteristics

**FIGURE 1:**
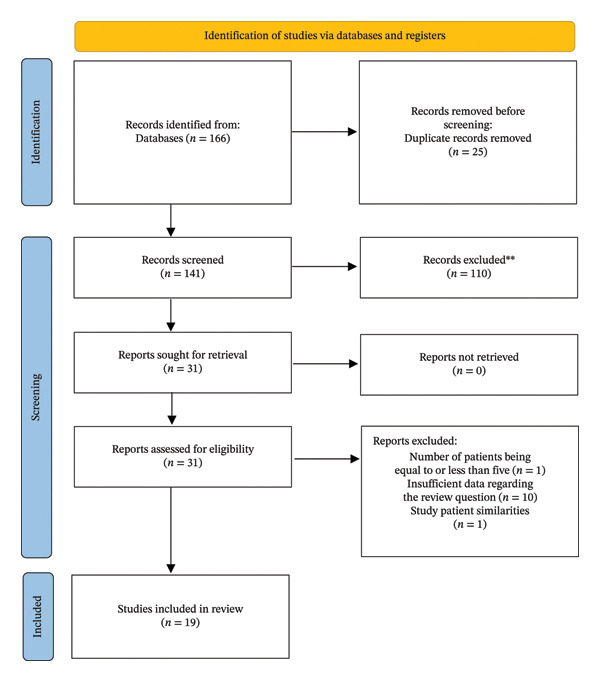
PRISMA flowchart. The primary reasons for record exclusion were duplicate records, nonoriginal or nonclinical studies, absence of SAAE as an intervention, insufficient outcome reporting, and inappropriate study design.

Nineteen peer‐reviewed studies, including 1469 patients with PA, met the eligibility criteria (Figure 1) [9–12, 14–28]. Of these, 1242 underwent SAAE, and 227 received MRAs. All studies were conducted in China, reflecting the current geographic concentration of available evidence. All studies were observational, and none were randomized clinical trials. All studies except one were prospective. Eighteen studies reported BP outcomes, sixteen reported biochemical outcomes, and fourteen assessed adverse events (Table 1).

**TABLE 1:** Characteristics of the included studies.

Author	Year	Country	Study design	AAE (*n*)	Medical treatment (*n*)	Age, AAE (mean ± SD)	Age, medical (mean ± SD)	Follow‐up period (month)	Primary outcomes reported			
									Blood pressure	Biochemical profile	Efficacy of AAE	Adverse events
Zhang et al. [21].	2020	China	Prospective	36		48.4 ± 12.5		6	+	+	+	+
Dong et al. [14].	2021	China	Prospective	41		41.0 ± 9.4		12	+	−	−	+
Zhao et al. [22].	2021	China	Prospective	26	25	NR		6	+	+	+	−
Zhou et al. [23].	2022	China	Prospective	74	66	54.2 ± 10.9	54.6 ± 14.7	12	+	+	+	+
Qiu et al. [18].	2023	China	Prospective	31		47 ± 11		12	+	+	+	+
Sun et al. [19].	2023	China	Prospective	52		45 ± 12		6	+	+	+	+
Zhou et al. [9].	2024	China	Prospective	29	30	55.52 ± 12.07	56.00 ± 15.77	6	+	+	−	+
Lai et al. [16].	2024	China	Prospective	182		48.9 ± 10.3		12	−	−	+	−
Mao et al. [17].	2024	China	Prospective	68		53.2 ± 13.1		12	+	+	+	+
Sun et al. [20].	2024	China	Prospective	93		47 ± 11		6	+	+	+	+
Li et al. [11].	2025	China	Prospective	38		51.2 ± 12.3		12	+	+	+	−
Ji et al. [10].	2025	China	Prospective	55		41.5 ± 8.8		6	+	+	−	−
Ji et al. [15].	2025	China	Prospective	94		51.1 ± 11.1		24	+	+	+	+
Wang et al. [12].	2025	China	Retrospective	93	53	50.98 ± 12.25	51.49 ± 11.92	12	+	−	+	+
Zhou et al. [24].	2025	China	Prospective	35	33	49.5 ± 7.4	48.9 ± 8.6	6	+	+	−	+
Khan et al. [25].	2025	China	Prospective	42	20	53.2 ± 13.1	51.5 ± 12.1	12	+	+	+	+
Shuai et al. [27].	2026	China	Prospective	129		51.30 ± 10.60		25	+	+	+	+
Zhoufei et al. [28].	2026	China	Prospective	25		51.88 ± 8.89		6.92	+	+	+	+
Liang et al. [26].	2026	China	Prospective	99		48.53 ± 10.20		6	+	+	−	−

### 3.2. Risk of Bias Assessment

Most domains were judged to be of low risk in the majority of studies (Supporting Figure 1). The one consistent problem was bias due to deviations from intended interventions, which was high risk in all studies because SAAE patients commonly continued antihypertensive medications during follow‐up. This cointervention complicates attribution of BP changes solely to SAAE and likely biases effects in favor of greater improvement.

### 3.3. BP Reduction of SAAE

SAAE was associated with substantial reductions in both systolic and diastolic BP across all measurement settings. For SBP, the pooled decreases were −18.58 mmHg (*p* < 0.001) in home monitoring, −19.71 mmHg (*p* < 0.001) in office readings, and −14.57 mmHg (*p* < 0.001) in 24‐h ambulatory monitoring. DBP followed a similar gradient, with reductions of −10.02 mmHg (*p* < 0.001), −10.91 mmHg (*p* < 0.001), and −8.55 mmHg (*p* < 0.001), respectively (Figure 2).

FIGURE 2Forest plots showing (A) SBP change after SAAE across different measurement settings, and (B) DBP change after SAAE across different measurement settings.

**Figure:**
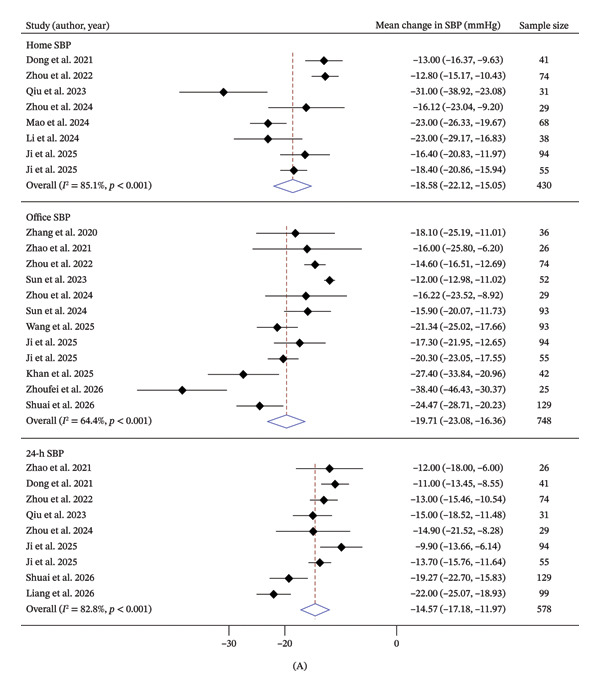

**Figure:**
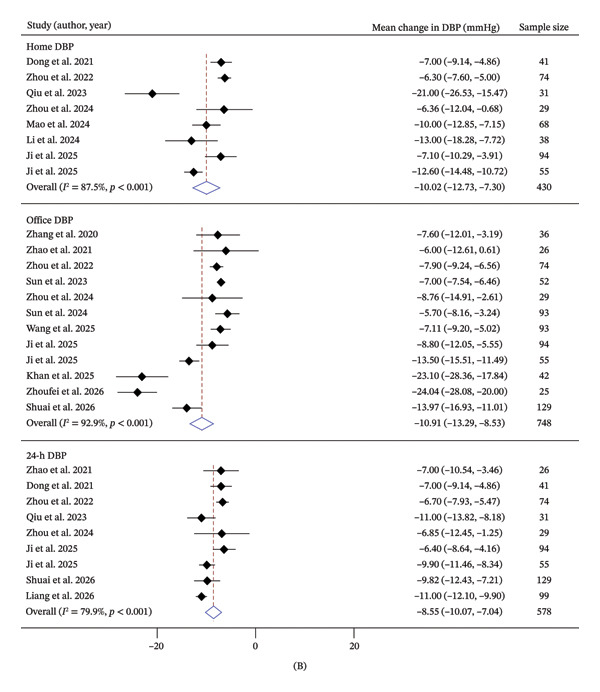

Alongside BP reduction, SAAE significantly decreased antihypertensive drug requirements (pooled DDD change = −0.52; CI: −0.81 to −0.23; *p* < 0.001), corresponding to roughly half a standard daily dose, a moderate but clinically relevant reduction in treatment burden (Supporting Figure 2).

MRAs alone also produced significant BP reductions, with pooled decreases of −15.50 mmHg in office SBP, −10.30 mmHg in 24‐h SBP, −7.82 mmHg in office DBP, and −5.34 mmHg in 24‐h DBP (Supporting Figure 3). Statistical comparisons between the two approaches showed only trivial numerical differences, none of which reached significance (home SBP: *p* = 0.410; office SBP: *p* = 0.213; 24 h SBP: *p* = 0.398; home DBP: *p* = 0.417; office DBP: *p* = 0.260; 24 h DBP: *p* = 0.303). Taken together, these findings confirm that both SAAE and MRAs achieve substantial improvements in BP, with no clear superiority of one method over the other (Supporting Figure 4). No evidence of publication bias was detected, and sensitivity analysis identified no influential studies, thereby further reinforcing the robustness of the findings.

### 3.4. Biochemical Outcomes and Comparative Effects of SAAE

SAAE was associated with favorable improvements across several biochemical markers of mineralocorticoid activity. Serum potassium increased significantly after SAAE (+0.34 mmol/L; *p* < 0.001), with a comparable rise observed after MRAs (+0.48 mmol/L; *p* < 0.001). Aldosterone levels declined substantially with SAAE (−46.72 ng/dL; *p* < 0.001), whereas MRAs produced an increase (+30.24 ng/dL; *p* < 0.001). Renin activity rose following both treatments, with a more pronounced increase after SAAE (1.49; *p* = 0.016) than after MRAs (1.99; *p* = 0.287). The ARR fell markedly after SAAE (−32.64; *p* < 0.001) but only modestly after MRAs (−14.80; *p* < 0.001). These biochemical effects of SAAE and MRAs are summarized in Figure 3.

FIGURE 3Forest plots showing changes in biochemical metrics after SAAE and medical treatment: (A) serum potassium, (B) serum aldosterone, (C) serum renin, and (D) serum ARR.

**Figure:**
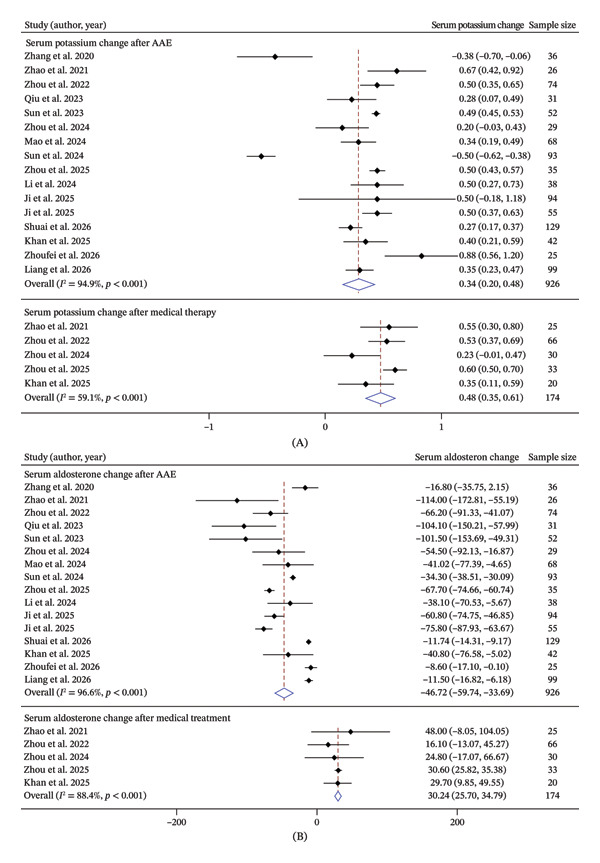

**Figure:**
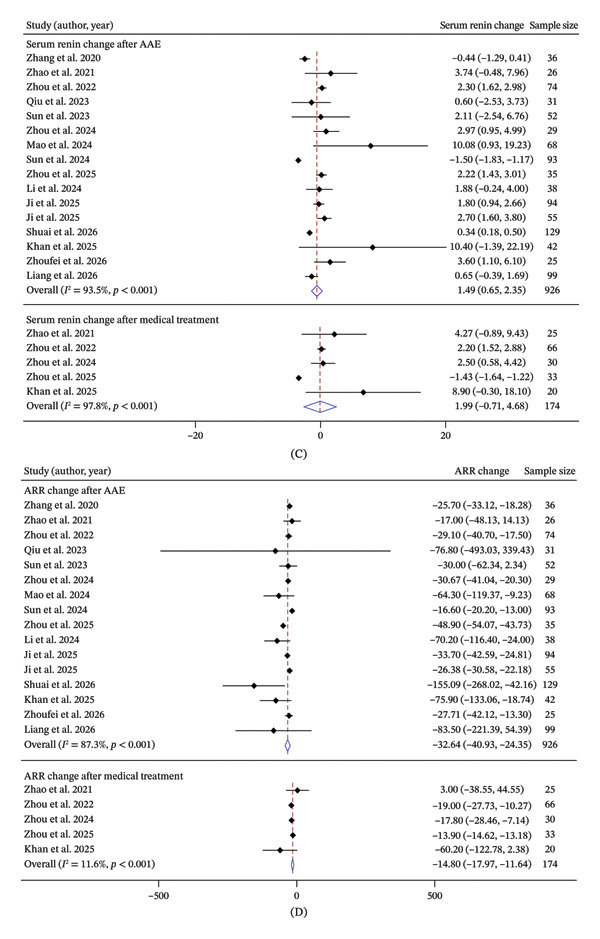

Comparisons between the two treatments showed no significant differences in potassium (*p* = 0.265), renin (*p* = 0.312), or ARR (*p* = 0.013). Taken together, both SAAE and MRAs improve the biochemical profile of PA, with SAAE showing numerically larger but not statistically significant advantages (Supporting Figure 5).

### 3.5. Efficacy of SAAE

Across the included studies, the pooled proportion of patients achieving a complete clinical response after SAAE was 27%, while an additional 43% achieved a partial clinical response (Figure 4A). Taken together, these findings indicate that the majority of patients undergoing SAAE experience measurable improvement in BP control, although complete normalization without antihypertensive medication is less common.

FIGURE 4(A) Pooled proportions of complete and partial clinical success after SAAE (PASO criteria). (B) Pooled proportions of complete and partial biochemical success after SAAE (PASO criteria).

**Figure:**
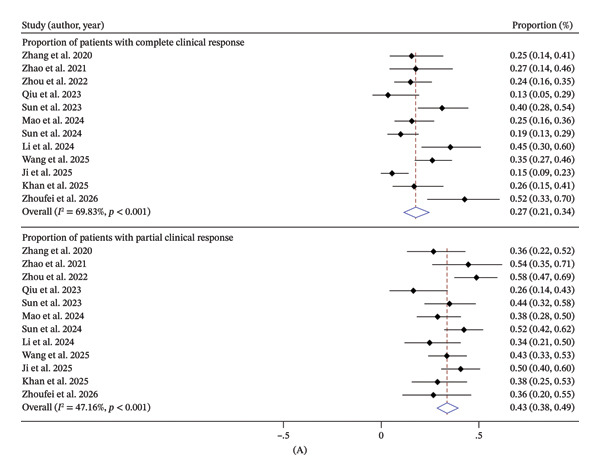

**Figure:**
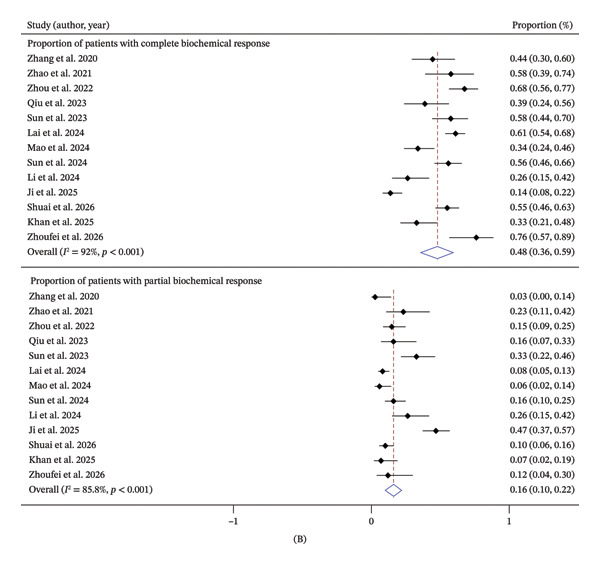

The pooled proportion of patients achieving a complete biochemical response after SAAE was 48%, while an additional 16% achieved a partial biochemical response (Figure 4B). These findings indicate that nearly two‐thirds of patients undergoing SAAE experience at least some normalization of mineralocorticoid activity, with complete biochemical cure in almost half.

### 3.6. Safety

Across the included studies, adverse events following SAAE were mostly minor, transient, and manageable with standard care. The most frequent events were postprocedural pain (back, flank, abdominal, or puncture‐site pain) (*n* = 313, 37.17%) and low‐grade fever (*n* = 72, 8.55%), both of which typically resolved with NSAID therapy. Labile BP during the procedure was also relatively common (*n* = 143, 16.98%) but was effectively controlled in most cases with nitroprusside. Less frequently reported events include nausea/vomiting (*n* = 57, 6.76%), dizziness with or without hypotension (*n* = 27, 3.21%), abdominal distension (*n* = 28, 3.33%), pleural effusion without complication (*n* = 7, 0.83%), hematoma (*n* = 3, 0.36%), diarrhea (*n* = 2, 0.24%), pruritus (*n* = 1, 0.12%), transient numbness of the puncture‐side limb (*n* = 7, 0.83%), transient elevation of pancreatic enzyme levels (*n* = 6, 0.71%), and headache (*n* = 8, 0.95%). Notably, pleural effusion and numbness resolved spontaneously without intervention.

Arrhythmias were reported in 93 patients (11.05%), almost all occurring during the procedure rather than afterward. These included ventricular tachycardia, ventricular premature beats, atrial premature beats, atrial tachycardia, atrial flutter, and atrial fibrillation. In the majority of cases, arrhythmias were self‐limiting or managed successfully without long‐term complications.

Serious adverse events were uncommon. Hypertensive crises occurred in five patients (0.59%), all during or immediately after the procedure, and were managed with appropriate antihypertensive therapy. Major cardiovascular or neurologic complications were uncommon and reported only sporadically, including stroke (*n* = 5, 0.59%), sinus arrest (*n* = 2, 0.24%), postprocedural heart failure (*n* = 1, 0.12%), and myocardial infarction (*n* = 2, 0.24%). These were noted after completion of the embolization rather than during the intervention.

Overall, these findings suggest that SAAE carries a predictable profile of minor adverse events, most of which are easily treated or self‐resolving. Major complications are uncommon, underscoring the procedure’s relative safety in appropriately selected patients.

## 4. Discussion

The present systematic review and meta‐analysis offers evidence supporting SAAE as a relatively safe and effective treatment for PA. By synthesizing data from nineteen observational studies encompassing 1242 patients, this analysis demonstrates that SAAE delivers clinically meaningful BP reductions, substantial improvements in mineralocorticoid biochemical profiles, and a favorable safety profile.

SAAE achieves robust BP control across settings. The pooled decrease in office systolic BP was −19.71 mmHg and that in diastolic BP was −10.91 mmHg. Moreover, home and ambulatory BP improvements after SAAE (−18.58 mmHg and −14.57 mmHg in SBP; −10.02 mmHg and −8.55 mmHg in DBP) underscore consistent efficacy across real‐world monitoring modalities. These outcomes compare favorably with those achieved by MRAs alone (−15.50 mmHg office SBP; −7.82 mmHg office DBP), with no significant differences in head‐to‐head comparisons, confirming that embolization attains parity with pharmacotherapy in BP reduction.

Beyond BP control, SAAE exerts distinctive biochemical advantages by directly ablating aldosterone‐producing tissue. Our pooled analysis revealed a striking decline in plasma aldosterone (−46.72 ng/dL) and a marked reduction in ARR (−32.64), effects not paralleled by MRAs, which block receptor activity without halting hormone secretion. The biochemical normalization after SAAE supports its mechanistic superiority in addressing the underlying pathophysiology of PA, potentially mitigating aldosterone‐mediated end‐organ damage more effectively than receptor blockade alone.

Clinically, a complete clinical response (normotension without antihypertensives) was achieved in 27% of SAAE patients, with an additional 43% attaining a partial response. These rates echo those observed after surgical adrenalectomy (complete success 27% in unilateral disease) and surpass outcomes reported in bilateral cohorts treated medically, highlighting SAAE’s capacity to bridge therapeutic gaps for both unilateral and bilateral PA [29]. Complete biochemical success was attained in 48% of patients, underscoring the procedure’s ability to restore hormonal homeostasis.

Several factors underscore the clinical utility of SAAE. First, it offers a one‐time intervention, reducing reliance on lifelong MRAs, which are associated with poor adherence due to gynecomastia, menstrual irregularities, and hyperkalemia. Second, SAAE expands options for medically complex or surgery‐averse patients, including those with bilateral disease who would otherwise face indefinite pharmacotherapy. Third, SAAE facilitates precision medicine by targeting aberrant adrenal segments guided by adrenal vein sampling, optimizing efficacy while sparing normal tissue. In addition, the procedure’s minimally invasive nature permits rapid recovery and outpatient management in many centers.

The safety profile of SAAE is characterized predominantly by minor, self‐limiting adverse events, namely, postembolization syndrome with postprocedural pain (37.17%) and low‐grade fever (8.55%). The incidence of serious complications, including hypertensive crises (0.59%), stroke (0.59%), and myocardial infarction (0.24%), remained exceedingly low, reinforcing the procedure’s relative safety in experienced centers. Notably, no severe cortisol suppression or adrenal insufficiency was reported, distinguishing SAAE from nonselective ablation techniques and preserving endogenous adrenal function [25]. Although their rarity is reassuring, these events highlight the importance of careful patient selection, vigilant intraprocedural hemodynamic monitoring, and immediate access to intensive medical management to ensure the safety of this promising therapy.

Future investigations of SAAE should prioritize large‐scale, multicenter randomized trials with standardized embolization protocols and long‐term follow‐up to rigorously compare SAAE against adrenalectomy and optimized MRA regimens, thereby defining its optimal role in the therapeutic sequence for PA. Integration of advanced imaging modalities such as three‐dimensional rotational angiography and fusion imaging may enhance target precision and enable assessment of perfusion changes postembolization, facilitating procedural refinement and identification of predictors of biochemical and clinical response. Additionally, parallel research should explore the molecular and genetic underpinnings of aldosterone‐producing adenomas and hyperplasia to identify biomarkers predictive of embolization success, potentially informing patient selection and personalized treatment algorithms. Furthermore, comprehensive evaluation of long‐term cardiovascular and renal outcomes, including cardiac remodeling and glomerular filtration rate preservation, will elucidate whether SAAE’s source‐control mechanism translates into durable end‐organ protection beyond BP normalization.

Several limitations warrant consideration. First, the evidence base is predominantly derived from observational studies, which constrains external validity and introduces risks of selection and performance bias. In addition, long‐term effects of SAAE on end‐organ outcomes (e.g., cardiac and renal remodeling) remain unknown; the technique is relatively new, and such endpoints have not yet been systematically evaluated. Furthermore, subgroup analyses stratified by the type of PA (unilateral versus bilateral dominant aldosterone secretion) were not feasible. Most included studies enrolled mixed populations of patients with both unilateral and bilateral disease and did not report outcomes separately according to lateralization status. This limits our ability to identify the optimal target population for SAAE and should be addressed in future studies. These gaps highlight the need for adequately powered, multicenter, prospective comparative studies with standardized outcomes and extended follow‐up.

## 5. Conclusion

SAAE is a relatively safe and effective treatment that lowers BP, reduces medication burden, and improves biochemical markers in PA by inhibiting aldosterone secretion. For patients who are not surgical candidates, who decline adrenalectomy, or who cannot tolerate or adhere to MRAs, SAAE provides a credible, patient‐centered option for disease control.

## Funding

The authors declare that no funds, grants, or other support were received during the preparation of this manuscript.

## Ethics Statement

This article does not contain any studies with human participants or animals performed by any of the authors.

## Consent

The authors have nothing to report.

## Conflicts of Interest

The authors declare no conflicts of interest.

## Supporting Information

Additional supporting information can be found online in the Supporting Information section.

## Supporting information


**Supporting Information 1** Supporting Information 1: Open Science Framework (OSF) registration record and protocol details.

**Supporting Information 2** Supporting Information 2: PRISMA 2020 checklist and PRISMA‐S checklist.

**Supporting Information 3** Supporting Information 3: Full electronic search strategies for all databases (PubMed, Web of Science, Embase, and Cochrane Library).

**Supporting Information 4** Supporting Figure 1. Risk of bias assessment based on ROBINS‐I. Supporting Figure 2: Forest plot of change in antihypertensive drug burden expressed as defined daily dose (DDD) after SAAE. Supporting Figure 3: Forest plots of blood pressure changes after MRA therapy (office and 24‐h ambulatory measurements). Supporting Figure 4: Forest plot of the mean difference between AAE and medical treatment effect on BP. Supporting Figure 5: Forest plot of the mean difference between AAE and medical treatment effect on biochemical metrics.

## Data Availability

All data used in this systematic review and meta‐analysis were derived from previously published studies and are available within the included articles and their supporting information.
