# Supplementary material for: Beyond Drugs and Surgery: Superselective Adrenal Artery Embolization Redefines Primary Aldosteronism Management—A Systematic Review and Meta‐Analysis
Source: Int J Endocrinol. 2026 Jul 22;2026:7534774. doi: 10.1155/ije/7534774 (PMC13389810; doi:10.1155/ije/7534774)
Supplement: Supplementary file 1 — Supporting Information 1 Supporting Information 1: Open Science Framework (OSF) registration record and protocol details. [file IJE-2026-7534774-s001.docx]

Effectiveness and Safety of Adrenal Artery Embolization in Patients with Primary Aldosteronism

1. Review question:

To evaluate the effectiveness and safety of adrenal artery embolization in patients with primary aldosteronism

2. Searches:

Two reviewers (A.M. and K.D.) developed their search syntax independently. In addition, EMTREE and MeSH keywords were checked. The finalized search syntax will be conducted on August 10, 2025, and all eligible studies will be included. We will search PubMed, Web of Science, and Embase databases. The reference lists of relevant articles will be reviewed to guarantee that no publications are missed. All searched studies will be imported into EndNote software for screening. Afterward, duplicate studies will be removed.

(Hyperaldostero* OR Conn OR Conns OR Conn's OR Aldostero*) AND (embo* OR ablat*)

3. Types of studies to be included:

Case-control, cohort, cross-sectional, and clinical trials with more than five patients will be included.

4. Condition or domain being studied:

Primary aldosteronism represents one of the most prevalent forms of secondary hypertension, affecting approximately 5-15% of hypertensive patients. The condition is characterized by excessive autonomous aldosterone secretion from the adrenal glands, leading to hypertension, hypokalemia, and increased cardiovascular morbidity compared to essential hypertension. Traditional management approaches have included mineralocorticoid receptor antagonists such as spironolactone and eplerenone for medical therapy, and laparoscopic adrenalectomy for unilateral aldosterone-producing adenomas.

Adrenal artery embolization has emerged as a minimally invasive alternative treatment modality, particularly for patients who are not suitable candidates for surgery or who refuse surgical intervention. The procedure involves transcatheter injection of embolic agents, typically absolute ethanol, into the adrenal arteries to ablate hyperfunctioning adrenal tissue. Despite growing clinical interest and reported efficacy in individual studies, comprehensive evidence regarding the effectiveness and safety of adrenal artery embolization remains limited.

Recent studies have demonstrated the efficacy of adrenal artery embolization in reducing blood pressure and correcting biochemical abnormalities associated with primary aldosteronism. However, direct comparative studies between adrenal artery embolization and medical therapy are sparse, and the relative benefits, risks, and long-term outcomes of these treatment approaches have not been systematically evaluated. Given the increasing utilization of adrenal artery embolization and the need for evidence-based treatment recommendations, a comprehensive systematic review and meta-analysis assessing this therapeutic modality is warranted.

This systematic review and meta-analysis aims to determine the effectiveness and safety of adrenal artery embolization in patients with primary aldosteronism. The primary objective is to evaluate comparative clinical and biochemical success rates. Secondary objectives include assessment of blood pressure control, changes in biochemical parameters, adverse events, quality of life measures, and long-term cardiovascular outcomes.

5. Participants/population:

Participants must be adults (18 years or older) with confirmed diagnosis of primary aldosteronism based on established diagnostic criteria, including elevated aldosterone-to-renin ratio and confirmatory testing where appropriate. Studies focusing exclusively on other forms of secondary hypertension or healthy volunteers will be excluded.

6. Intervention(s), exposure(s):

The intervention of interest is adrenal artery embolization performed via transcatheter approach using any embolic agent, including absolute ethanol, anhydrous ethanol, or other approved embolic materials. Both unilateral and bilateral embolization procedures will be included. Studies describing percutaneous ablation techniques other than arterial embolization will be excluded.

7. Comparator(s)/control:

The comparator will be medical therapy for primary aldosteronism, primarily consisting of mineralocorticoid receptor antagonists such as spironolactone, eplerenone, or other approved agents if available and reported. Studies comparing adrenal artery embolization with surgical adrenalectomy alone will be excluded unless medical therapy arms are also included.

8. Main outcome:

Primary outcomes will include clinical success (defined as blood pressure normalization or significant reduction with or without antihypertensive medications) and biochemical success (defined as normalization of aldosterone, renin, and aldosterone-to-renin ratio).

9. Additional outcome(s):

Secondary outcomes will encompass blood pressure measurements (office, home, and 24-hour ambulatory monitoring), serum potassium levels, plasma aldosterone concentrations, plasma renin activity or concentration, aldosterone-to-renin ratio, medication requirements, adverse events, quality of life measures, and long-term cardiovascular outcomes.

10. Data extraction (selection and coding):

Two reviewers, K.D., M.Z., will separately extract data from the included studies in order to respond to the review question. In the following phase, the files will be compared after the data has been entered into an Excel spreadsheet. A third reviewer, A.Mo, will be involved if disagreements cannot be settled.

11. Strategy for data synthesis:

The analysis will be conducted using STATA version 17.0. A random-effects model will

be used. The I2 ≥ 50% will be considered a high statistical heterogeneity

.

12. Contact details for further information:

*Anonymized for peer review*

13. Organizational affiliation of the review:

*Anonymized for peer review*

14. Type and method of review:

Systematic review and meta-analysis

15. Anticipated or actual start date:

August 10, 2025

16. Anticipated completion date:

November 10, 2025

17. Funding sources/sponsors:

None

18. Conflicts of interest:

None
