# Supplementary material for: Beyond Drugs and Surgery: Superselective Adrenal Artery Embolization Redefines Primary Aldosteronism Management—A Systematic Review and Meta‐Analysis
Source: Int J Endocrinol. 2026 Jul 22;2026:7534774. doi: 10.1155/ije/7534774 (PMC13389810; doi:10.1155/ije/7534774)
Supplement: Supplementary file 3 — Supporting Information 3 Supporting Information 3: Full electronic search strategies for all databases (PubMed, Web of Science, Embase, and Cochrane Library). [file IJE-2026-7534774-s003.docx]

Supplemental Material 3. Literature Search Syntax

| Searched database | Search syntax |
| --- | --- |
| PubMed and Cochrane library | (Hyperaldostero* OR Conn OR Conns OR Conn's OR Aldostero*) AND (embo* OR ablat*) |
| Web of Science and Embase | (Hyperaldostero* OR Conn OR Conns OR Conn's OR Aldostero*) AND (embo* OR ablat*) |

Ovid was used to concurrently search all databases.
