# Supplementary material for: Beyond Drugs and Surgery: Superselective Adrenal Artery Embolization Redefines Primary Aldosteronism Management—A Systematic Review and Meta‐Analysis
Source: Int J Endocrinol. 2026 Jul 22;2026:7534774. doi: 10.1155/ije/7534774 (PMC13389810; doi:10.1155/ije/7534774)

Supplementary Figure 1. Risk of bias assessment based on ROBINS-I.


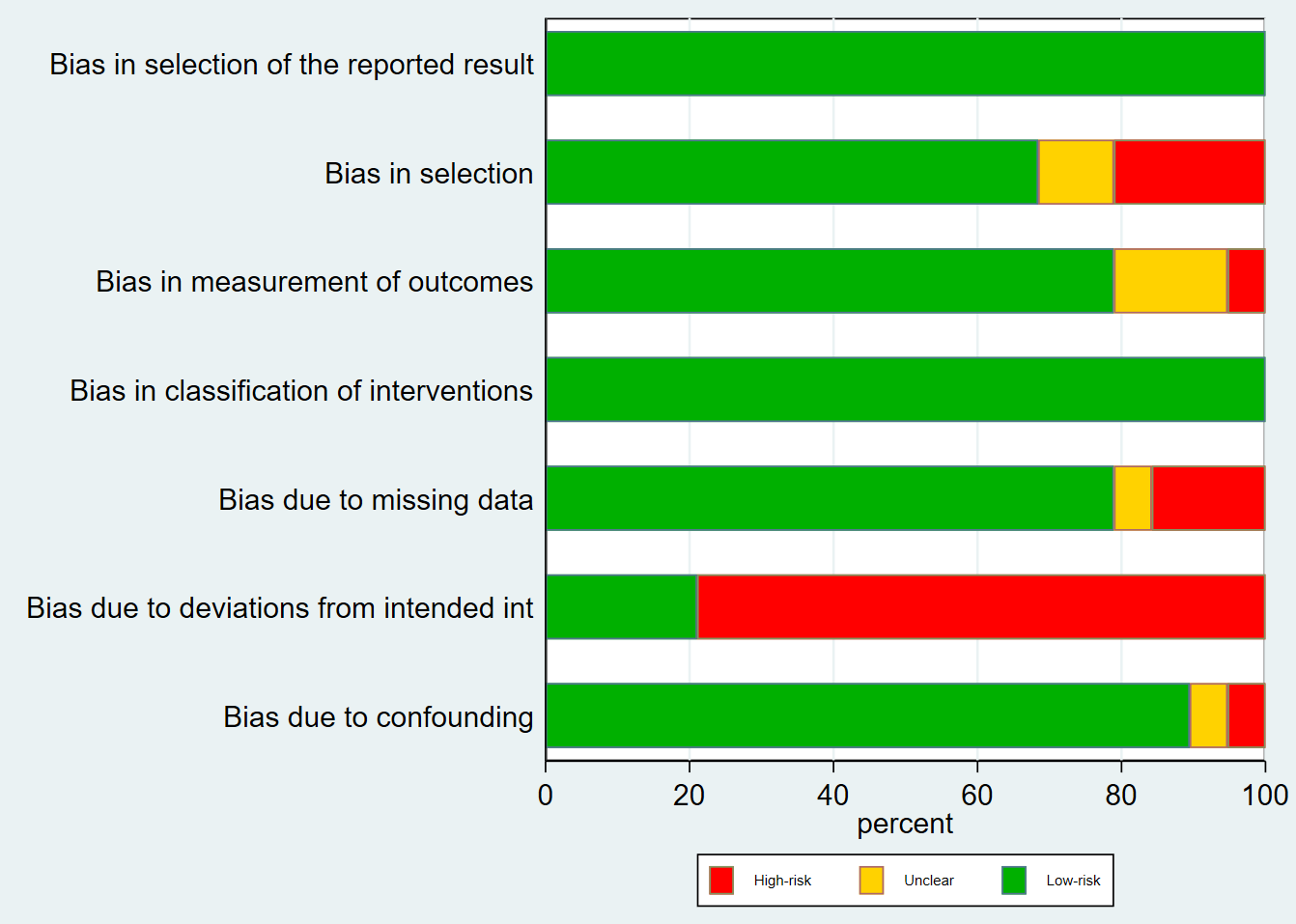


Supplementary Figure 2: Forest plot showing the effect of AAE on the defined daily dose:


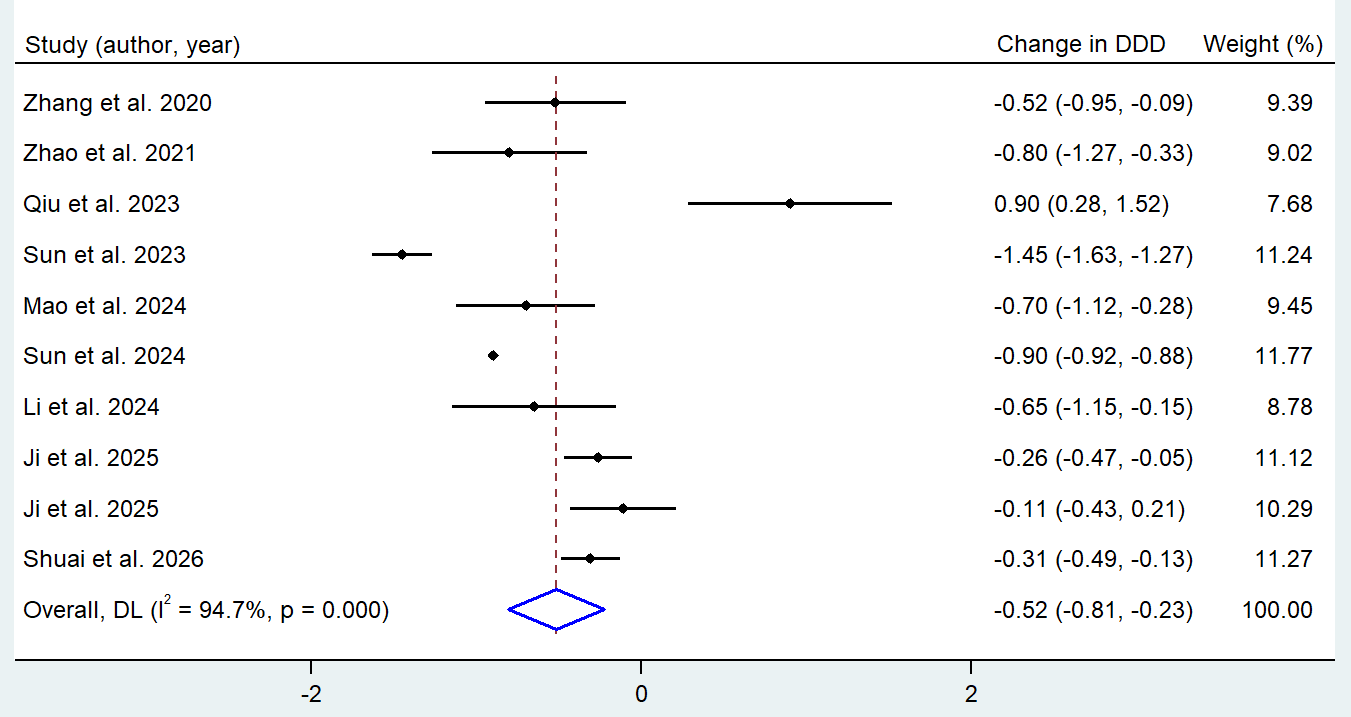


Supplementary Figure 3: Forest plot showing the pooled effect of medical treatment on patients' BP (only two studies reported home BP, therefore excluded from this analysis):


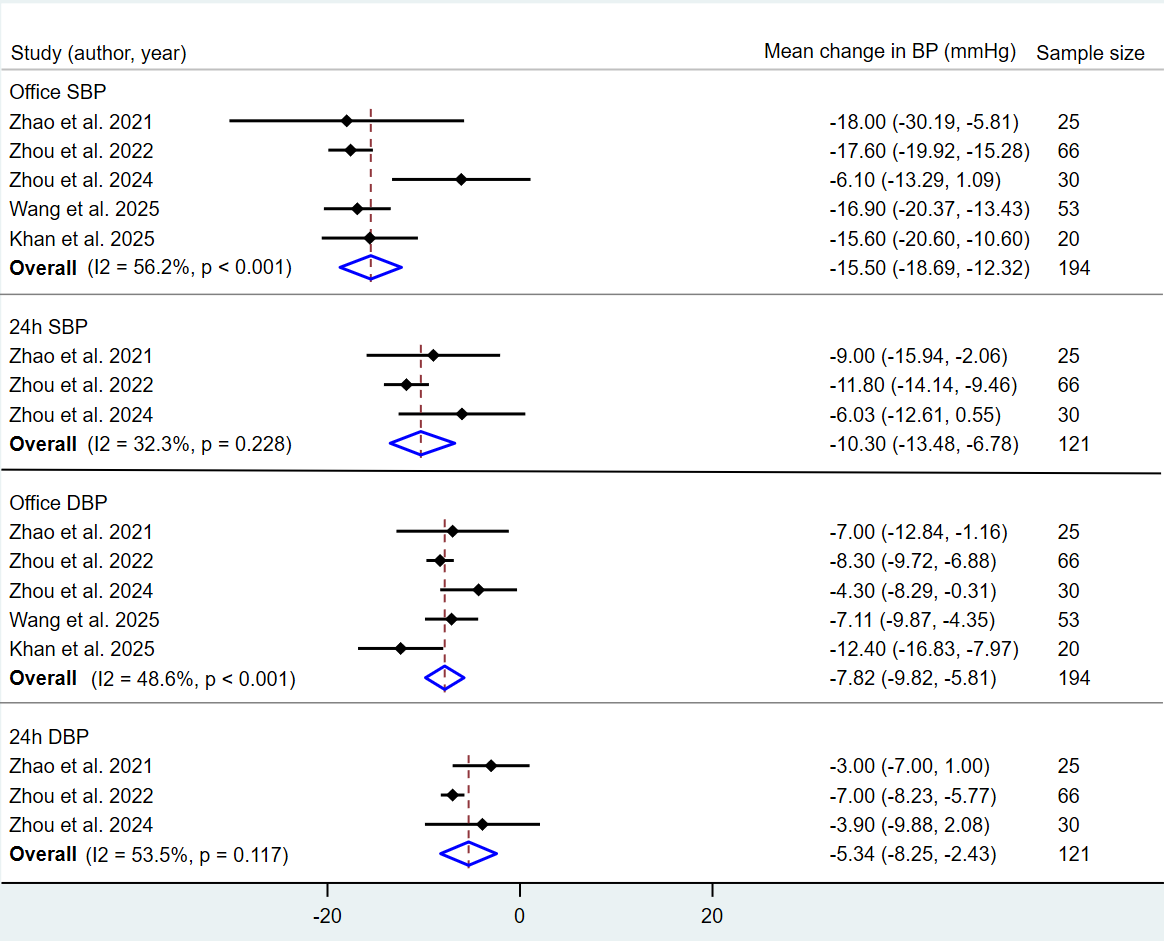


Supplementary Figure 4: Forest plot of the mean difference between AAE and medical treatment effect on BP


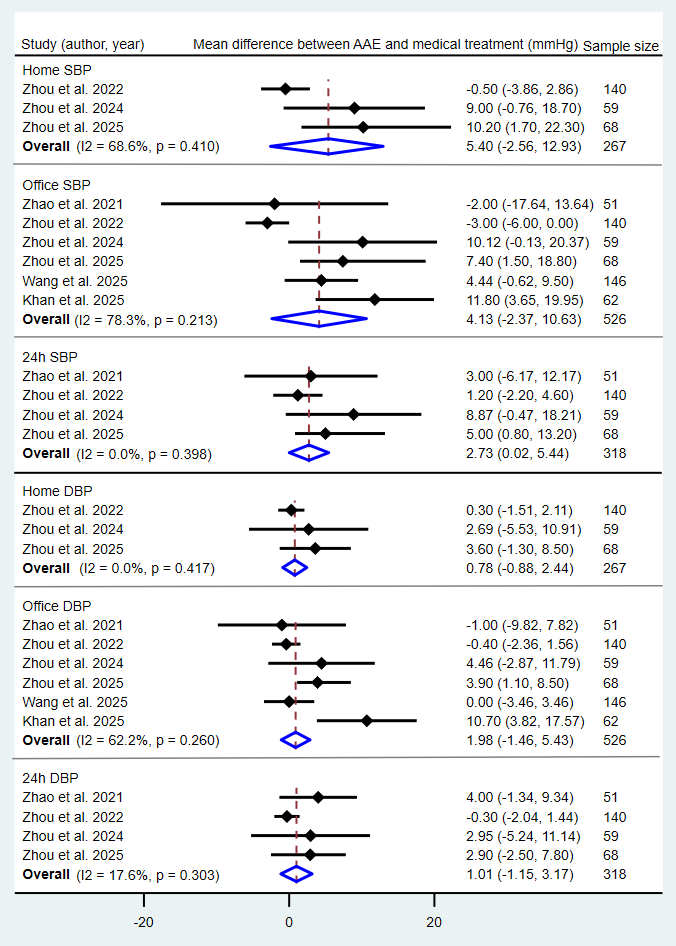


Supplementary Figure 5: forest plot of the mean difference between AAE and medical treatment effect on biochemical metrics


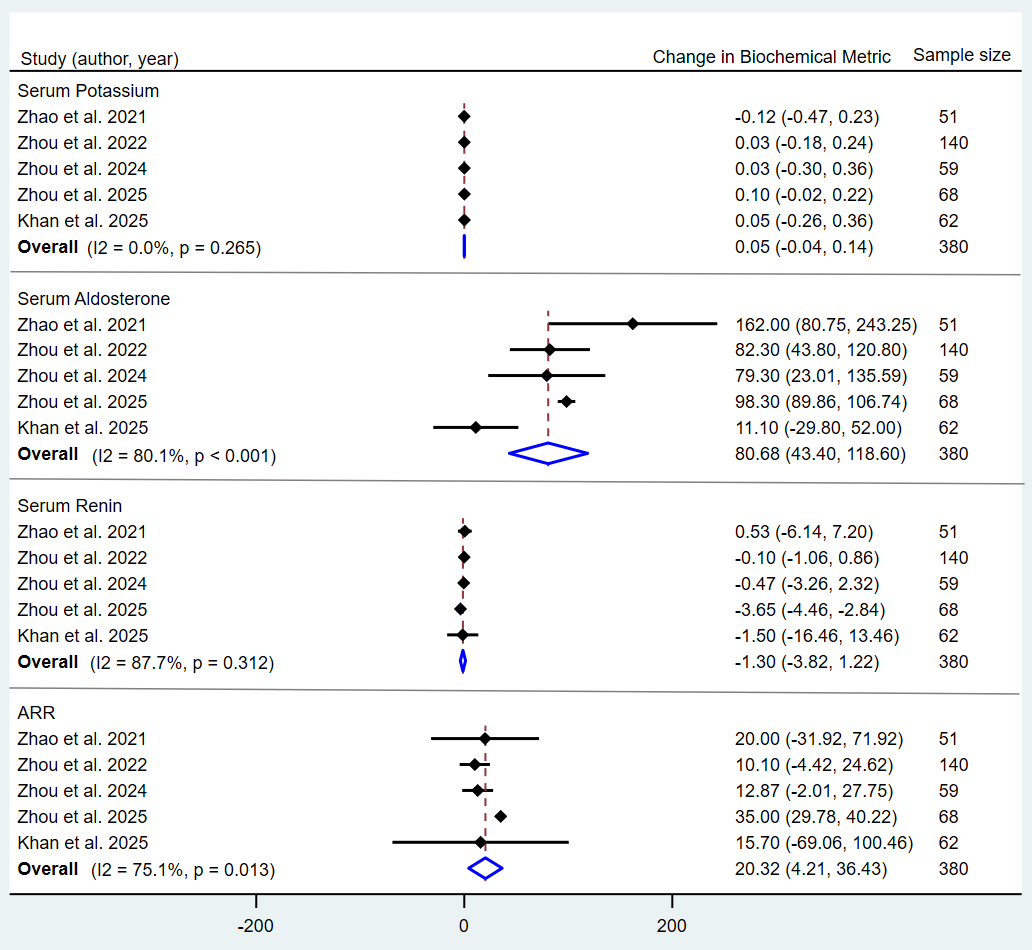

Supplement: Supplementary file 4 — Supporting Information 4 Supporting Figure 1. Risk of bias assessment based on ROBINS‐I. Supporting Figure 2: Forest plot of change in antihypertensive drug burden expressed as defined daily dose (DDD) after SAAE. Supporting Figure 3: Forest plots of blood pressure changes after MRA therapy (office and 24‐h ambulatory measurements). Supporting Figure 4: Forest plot of the mean difference between AAE and medical treatment effect on BP. Supporting Figure 5: Forest plot of the mean difference between AAE and medical treatment effect on biochemical metrics. [file IJE-2026-7534774-s004.docx]
